# Multiple Trichoepithelioma Syndrome: A Case Report

**DOI:** 10.7759/cureus.42930

**Published:** 2023-08-04

**Authors:** Frederico P Guerreiro, Ana I Martins, Joana Costa e Silva, Nelson Teixeira, José N Ramos

**Affiliations:** 1 Plastic, Reconstructive, and Maxillofacial Surgery, Centro Hospitalar de Lisboa Ocidental, Lisbon, PRT

**Keywords:** brooke-spiegler, skin nodule, cylindroma, spiradenoma, trichoepithelioma

## Abstract

Multiple trichoepithelioma syndrome is a rare entity, and little is known about its epidemiological features. Patients usually present with multiple nonsuspicious skin lesions. Surgical excision is the mainstay of treatment, and diagnosis is usually made after the first pathology report. Once the diagnosis is established, patients are kept under clinical surveillance, and surgery is performed again if tumor burden and/or size justifies it.

The authors present a male patient who presented to our outpatient clinic for the first time in 36 years without any relevant medical history, medication, or allergies. The patient had complaints of multiple skin lesions spreading across the head and neck regions. Surgical excision of the affected area and resurfacing using local advancement flaps were performed. Pathology reports were always consistent with trichoepitheliomas. No pathology of spiradenoma or cylindroma was ever reported.

Usually, tumors are small enough for simple excision and primary closure. However, in the presented case, the size of the tumor and the involvement of central facial aesthetic units demanded a more complex approach.

## Introduction

Multiple trichoepithelioma syndrome is a rare entity, and little is known about its epidemiological features. Patients usually present with multiple small smooth, nonulcerated, skin-colored papules that nonetheless may carry the burden of disfigurement, thus affecting them physically and psychologically. Surgical excision is the mainstay of treatment, and diagnosis is usually made after the first pathology report. Once the diagnosis is established, patients are kept under clinical surveillance, and surgery is performed again if tumor burden and/or size justifies it [1].

## Case presentation

**Figure 1:**
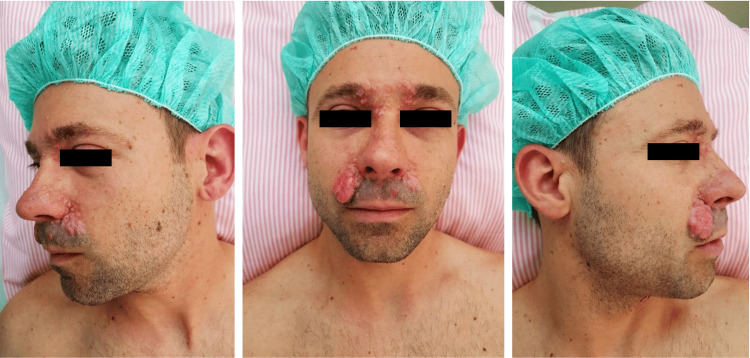
Patient’s preoperative presentation

The presented case report refers to a 36-year-old male patient who presented to our outpatient clinic for the first time without any relevant medical history, medication, or allergies and who had complaints of multiple smooth, nonulcerated, skin-colored papules spreading across multiple facial regions. Of note, the largest lesion involved the right nasolabial fold (Figure 1). Surgical excision of the affected area and resurfacing were performed using local advancement flaps. Evolution was favorable, with excellent aesthetic results. The pathology report revealed a trichoepithelioma.

Since then, the patient has kept follow-up at our outpatient clinic. During this time (around eight years), more smaller lesions appeared, mostly in the forehead and scalp regions, and periodical surgical excision of the most cumbersome lesions was performed. Pathology reports were always consistent with trichoepitheliomas. No pathology of spiradenoma or cylindroma was ever reported.

## Discussion

Trichoepithelioma is a benign epithelial tumor composed of follicular germinative cells resembling those seen in the embryologic buds of primitive folliculosebaceous units. It presents as a smooth, nonulcerated, skin-colored papule, sometimes with associated telangiectasias. It is usually located on the head and neck. Most cases occur in adults after the age of 40. Histologically, trichoepithelioma (cribriform trichoblastoma) is a biphasic neoplasm with histologic features of dual differentiation toward follicular germinative epithelium and specific follicular stroma. The characteristic histopathology demonstrates basaloid cells with peripheral palisading that are arranged in nests or cribriform patterns surrounded by dense stroma and fibroblasts. Areas with ductal differentiation and lymphocyte infiltration resembling spiradenoma have also been observed on biopsies from patients with multiple familial trichoepithelioma-1 (MFT-1). The presence of multiple trichoepitheliomas should raise suspicion of Brooke-Spiegler syndrome (BRSS). BRSS is an autosomal dominant disorder characterized by multiple benign skin tumors that develop from adnexal structures of the skin. Individuals with BRSS may develop several types of adnexal tumors, most commonly spiradenomas, cylindromas, and trichoepitheliomas. Phenotypic variants of BRSS include MFT-1, if only presenting with trichoepitheliomas, and familial cylindromatosis (FC), if only presenting with cylindromas. BRSS is a rare disorder; its exact incidence and prevalence are unknown. BRSS occurs in all ethnic groups and affects both females and males equally [2].

Brooke-Spiegler syndrome (BRSS) is caused by mutations within cylindromatosis (*CYLD*), a tumor suppressor gene encoding an enzyme that interacts with multiple substrates of the nuclear factor kappa B (NF-kB) signaling pathway and downregulates its activity. FC and MFT-1 are allelic disorders resulting from mutations in the same gene. These clinical variants are also known as *CYLD* cutaneous syndrome. Germline *CYLD* mutations are detected in approximately 80%-85% of individuals with classic BRSS and 40%-50% of individuals with MFT-1 [3,4]. Cutaneous neoplasms of BRSS also harbor a large spectrum of somatic mutations, representing both loss of heterozygosity and sequence alterations.

Clinical manifestations include both skin tumors and extracutaneous tumors. Cutaneous neoplasms in BRSS first manifest during childhood and early adolescence. These tumors gradually enlarge in size, continue to increase in number throughout the patient’s lifetime, and can be disfiguring. The burden of the disease is therefore both physical and psychological. In individuals with MFT-1, multiple trichoepitheliomas usually present as discrete, small, confluent, and skin-colored papules on the face, concentrating symmetrically around the nose and nasolabial folds. The tumors can also extend to the inner aspects of the eyebrows and lateral aspects of the cheeks [5]. The severity of MFT-1 varies, ranging from dozens to hundreds that cause disfigurement. Malignant transformation can occur in preexisting neoplasms in 5%-10% of the affected individuals. Rapid enlargement of a given lesion can be of concern for malignant transformation [6].

Individuals with BRSS may rarely develop extracutaneous tumors in the parotid or minor salivary glands. These tumors usually occur later in life, with most cases reported beyond the fourth decade.

BRSS is suspected in a patient presenting with multiple benign cutaneous adnexal tumors (cylindroma, spiradenoma, and/or trichoepithelioma) that occurred at an early age and with a family history of similar multiple tumors suggesting an autosomal dominant pattern of inheritance. Histologic evaluation of tissue biopsies from cutaneous tumors is required for the diagnosis of the specific tumor type. The presence of more than one type of adnexal tumor on the same tissue biopsy is suggestive of BRSS. The definitive diagnosis is based on the combination of the following: (1) early age of onset for skin neoplasms and progression of the disease over time, (2) a family history of similar neoplasms and inheritance pattern, (3) histopathologic findings on tissue biopsy, (4) association with tumors in the parotid or other minor salivary glands, and (5) genetic testing demonstrating a germline mutation in *CYLD*.

Genetic testing should be considered for individuals with multiple cylindromas, spiradenomas, or trichoepitheliomas alone or in combination. As some affected individuals may not develop skin tumors until the fourth decade, genetic testing in unaffected individuals with a family history of BRSS can help facilitate early diagnosis and family planning.

The differential diagnosis of solitary or multiple skin neoplasms on the head and neck includes basal cell carcinoma, primary cutaneous adenoid cystic carcinoma, cutaneous metastasis of adenoid carcinoma of the salivary gland or visceral organ carcinoma, syringoma, and trichofolliculoma. Multiple trichoepitheliomas can occur in or mimic other genetic syndromes, including Bazex-Dupré-Christol syndrome, Nevoid basal cell nevus syndrome, or Rombo syndrome.

Surgical removal of skin tumors is the mainstay of treatment. Since the tumors are benign in most cases, complete excision may not be necessary once the diagnosis is confirmed histologically. Surgical removal of benign neoplasms is mainly aimed at minimizing symptoms and improving function and cosmesis. Destructive therapies, including curettage, cryosurgery, electric cautery, and laser resurfacing, have also been used. Because BRSS patients usually develop multiple neoplasms throughout their lifetime, repeated procedures may be needed [7,8].

This is a rare case in our plastic surgery department, since, in most situations, no reconstructive techniques are required, as most tumors are small enough for simple excision and primary closure. However, in the presented case, the size of the tumor and the involvement of central facial aesthetic units demanded a more complex approach. As such, following tumor excision, local advancement flaps made it possible to place scars in inconspicuous locations. Since then, clinical surveillance has made it possible for the tumors to be removed early enough for primary closure.

## Conclusions

As physicians who treat skin and adnexal conditions on a daily basis, both dermatologists and plastic surgeons should have a comprehensive knowledge of even the rarest clinical entities. The presented clinical case is a reminder of such a fact.

Multiple trichoepithelioma syndromes, albeit rare, may have devastating implications on any affected individual, and appropriate diagnosis, treatment, and counseling are essential. In some cases, such as the presented one, plastic surgeons may be required for the resurfacing of large skin defects or reconstruction of delicate parts, such as the aesthetic subunits of the face, and minimizing the disfigurement that these lesions may imply.
